# Supplementary material for: Synthesis and Characterization of 1-Hydroxy-5-Methyltetrazole and Its Energetic Salts
Source: Molecules. 2025 Jun 27;30(13):2766. doi: 10.3390/molecules30132766 (PMC12251141; doi:10.3390/molecules30132766)
Supplement: Supplementary file 1 [file molecules-30-02766-s001.zip › molecules-3696149-supplementary.pdf]

Chemistry of 1-Hydroxy-5-Methyl Tetrazole and its energetic Salts

Lukas J. Eberhardt, Maximilian Benz, Jörg Stierstorfer, Thomas M. Klapötke*

Department of Chemistry, Inorganic Chemistry, Chair of Small Molecule and Energetic Materials Research, LMU Munich, Butenandtstr. 5-13, 81377 Munich

tmk@cup.uni-muenchen.de

Table of Contents

1. Experimental Procedures

2. X-Ray Diffraction

3. Computation

4. NMR Spectroscopy

5. Toxicity

6. References

1. Experimental Procedures

^{1}H and ^{13}C spectra were recorded on BRUKER AMX 400 instruments. Chemical shifts are referenced with respect to DMSO-d_6 ^{1}H-NMR = 2.50, ^{13}C-NMR = 39.52. The chemical shifts have been expressed in parts per million (ppm) and the coupling constants (J) are given in Hertz (Hz). The following abbreviations for the signal multiplicity were used: s = singlet, br s = broad singlet. Infrared spectra (IR) were recorded in the region 4000-400 cm^{-1} on a PERKIN ELMER Spectrum BX-59343 instrument with a SMITHS DETECTION DuraSamplIR II Diamond ATR sensor. High-resolution mass spectra (HR-MS) was recorded on a FINNIGAN MAT 95Q with an Ion-Trap MS/MS system or a THERMO FISHER Q EXACTIVE GC Orbitrap GC-MS/MS. Decomposition temperatures were measured via differential thermal analysis (DTA) with an OZM Research DTA 552-Ex instrument at a heating rate of 5 °C min^{-1} and in a range of room temperature to 400 °C. All sensitivities toward impact (IS) and friction (FS) were determined according to BAM (Bundesanstalt für Materialforschung und Prüfung) standards using a BAM drop hammer and a BAM friction apparatus by applying the 1 of 6 method.[S1]

CAUTION! Some investigated compounds are potentially explosive materials. Safety precautions and equipment (such as wearing leather coat, face shield, Kevlar sleeves, Kevlar gloves, earthed equipment and ear plugs) should be used during all manipulations.

5-Methyltetrazole (1)

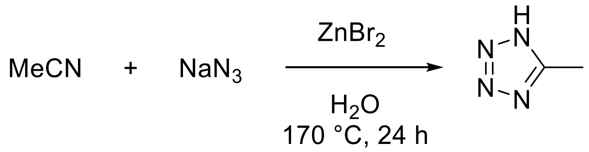

5-Methyltetrazole was synthesized according to a modified procedure by Sharpless *et. al*.[S2] Acetonitrile (16.9 mL, 320.0 mmol, 2.0 eq), sodium azide (10.4 g, 160.0 mmol, 1.0 eq) and zinc bromide (36.0 g, 160.0 mmol, 1.0 eq) were dissolved in water (160 mL) and transferred to a 350 mL pressure tube. The reaction flask was heated to 170 °C for 24 h. After cooling to room temperature, sodium hydroxide (16.0 g, 400.0 mmol, 2.5 eq) dissolved in 50 mL water was added, the precipitated $Zn(OH)_2$ was filtered off and washed with water (20 mL). The filtrate was acidified to pH = 1

using concentrated hydrochloric acid and saturated with magnesium sulfate. The aqueous layer was extracted with ethyl acetate (6 × 200 mL), dried over anhydrous sodium sulfate and concentrated under reduced pressure yielding 5-methyltetrazole (12.1 g, 143.9 mmol, 90%) as a white powder.

^{1}H NMR (DMSO-d_6, 400 MHz, ppm) δ = 15.94 (br s, 1H), 2.49 (s, 3H)

^{13}C NMR (DMSO-d_6, 101 MHz, ppm) δ = 152.7, 8.9.

The obtained analytical data is consistent with previously reported literature values.[S2]

1-Hydroxy-5-methyltetrazole (2)

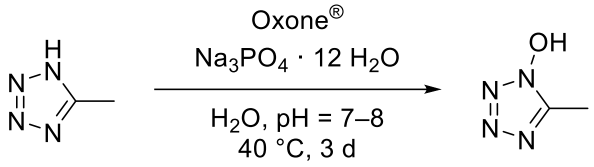

1-Hydroxy-5-methyltetrazole was synthesized according to a modified procedure by Klapötke *et al.*[S3] 5-Methyltetrazole (1.75 g, 20.8 mmol, 1 eq) was dissolved in water (60 mL) and sodium phosphate dodecahydrate was added to reach a pH value of 7. The mixture was heated to 40 °C. Over 1 h, Oxone (25.5 g, 83.2 mmol, 4 eq) and $Na_3PO_4 \cdot 12\ H_2O$ were added in alternating manner keeping the pH between 7–8. The suspension was stirred for 3 d at 40 °C. Afterwards, sulfuric acid (20%, 50 mL) was added until all solid was dissolved. The mixture was extracted with three portions of ethyl acetate (200 mL). The organic solvent was dried over anhydrous sodium sulfate and evaporated under reduced pressure. The crude product was recrystallized from acetone and *i*-hexane yielding 1-hydroxy-5-methyl tetrazole as colourless crystals (1.08 g, 10.8 mmol, 52%).

Sensitivities: Bam drop hammer: 3 J, **friction tester**: 160 N

DTA (5 ° min^{-1}): 146 °C (endo), 194 °C (dec).

Elem. Anal. ($C_2H_4N_4O$, 100,08 g mol^{-1}) calcd.: C 24.00, N 55.98, H 4.03%. Found: C 24.06, N 55.86, H 3.77%.

^{1}H NMR (400 MHz, DMSO-d_6) δ = 13.84 (br s, 1H), 2.43 (s, 3H).

^{13}C NMR (101 MHz, DMSO-d_6) δ = 145.8, 7.4.

HR-MS (ESI, 70 eV): [$C_2H_4N_4O$] calcd.: 99.0312 (M – H^+), found: 99.0312.

IR: 3919 (w), 3885 (w), 3818 (w), 3759 (w), 3665 (w), 3644 (w), 3458 (w), 3323 (w), 3272 (w), 3243 (w), 2948 (w), 2232 (m), 2200 (m), 2180 (m), 2166 (m), 2155 (w), 2044 (w), 2023 (w), 2004 (m), 1989 (w), 1619 (m), 1521 (s), 1396 (s), 1296 (s), 1272 (m), 1248 (s), 1137 (s), 1097 (s), 1035 (s), 996 (m), 946 (m), 746 (m), 717 (w), 681 (s), 654 (m), 518 (w), 480 (s), 425 (w), 414 (w), 404 (w).

Ammonium 5-methyltetrazol-1-olate (3)

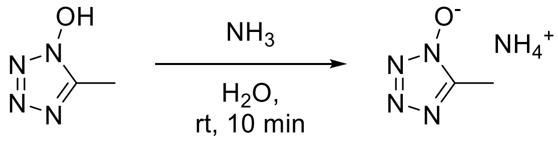

1-Hydroxy-5-methyltetrazole (250 mg, 2.5 mmol, 1 eq.) was dissolved in water (2 mL) and aqueous ammonia (2 M, 2 mL) was added. After stirring for 10 minutes the solution was transferred to a crystallizing dish yielding ammonium 5-methyltetrazol-1-olate as colorless crystals (290 mg, 2.48 mmol, 99%).

Sensitivities: Bam drop hammer: >40 J, **friction tester**: 360 N

DTA (5 ° min^{-1}): 166 °C (endo), 229 °C (dec).

Elem. Anal. ($C_2H_7N_5O$, 117.11 g mol^{-1}) calcd.: C 20.51, N 59.80, H 6.03%. Found: C 20.67, N 59.50, H 6.10%.

^{1}H NMR (400 MHz, DMSO-d_6) δ = 7.30 (br s, 4H), 2.14 (s, 3H).

^{13}C NMR (101 MHz, DMSO-d_6) δ = 140.3, 7.8.

IR: 3944 (w), 3734 (w), 3689 (w), 3574 (w), 3518 (w), 3151 (w), 2998 (m), 2852 (m), 2198 (w), 2167 (w), 2154 (w), 2004 (w), 1989 (w), 1913 (w), 1693 (w), 1532 (m), 1485 (m), 1440 (s), 1379 (m), 1238 (s), 1137 (m), 1112 (w), 1095 (m), 1040 (m), 1006 (w), 979 (w), 757 (m), 718 (w), 680 (m), 673 (m), 657 (w), 515 (m), 444 (w), 434 (w), 415 (w).

Hydroxylammonium 5-methyltetrazol-1-olate (4)

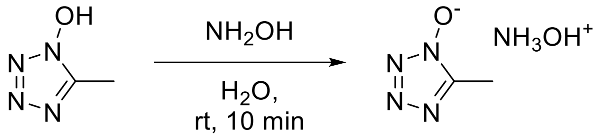

1-Hydroxy-5-methyltetrazole (200 mg, 2.0 mmol, 1 eq.) was dissolved in water (2 mL) and hydoxylamine (50 wt. %, 120 µL, 2.0 mmol, 1 eq.) was added. The water was evaporated under a stream of nitrogen, the crude product was recrystallized from ethanol yielding 5-methyltetrazol-1-olate as colorless crystals (169 mg, 1.3 mmol, 63%).

Sensitivities: Bam drop hammer: >40 J, **friction tester**: 288 N

DTA (5 ° min^{-1}): 139 °C (endo), 141 °C (dec).

Elem. Anal. ($C_2H_7N_5O$, 117.11 g mol^{-1}) calcd.: C 18.05, N 52.61, H 5.30%. Found: C 18.29, N 52.81, H 5.46%.

^{1}H NMR (400 MHz, DMSO-d_6) δ = 8.36 (br s, 4H), 2.21 (s, 3H).

^{13}C NMR (101 MHz, DMSO-d_6) δ = 142.6, 7.5.

IR: 3952 (w), 3857 (w), 3743 (w), 3620 (w), 3570 (w), 3483 (w), 2506 (s), 2232 (w), 2165 (w), 2147 (w), 2004 (w), 1962 (w), 1752 (w), 1639 (m), 1596 (m), 1522 (s), 1466 (m), 1433 (m), 1381 (m), 1244 (s), 1230 (s), 1132 (w), 1111 (w), 1042 (w), 1022 (w), 1001 (s), 921 (s), 758 (w), 718 (w), 682 (s), 510 (s), 448 (w), 434 (w).

Hydrazinium 5-methyltetrazol-1-olate (5)

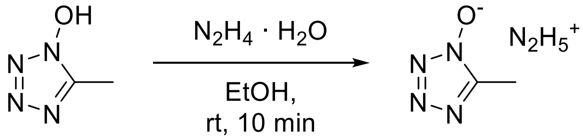

1-Hydroxy-5-methyltetrazole (250 mg, 2.5 mmol, 1 eq.) was dissolved in hot EtOH (2 mL). Hydrazine monohydrate (121 µL, 2.5 mmol, 1 eq.) was added and the solution was stirred for 10 min. Upon cooling hydrazinium 5-methyltetrazol-1-olate precipitated as colourless needles (228 mg, 1.7 mmol, 69%).

Sensitivities: Bam drop hammer: >40 J, **friction tester**: 360 N

DTA (5 ° min^{-1}): 105 °C (endo), 224 °C (dec).

Elem. Anal. ($C_2H_8N_6O$, 132.13 g mol^{-1}) calcd.: C 18.18, N 63.61, H 6.10%. Found: C 18.47, N 63.68, H 6.13%.

^{1}H NMR (400 MHz, DMSO-*d*$_6$) δ = 6.67 (br s, 5H), 2.17 (s, 3H).

^{13}C NMR (101 MHz, DMSO-*d*$_6$) δ = 140.4, 7.9.

IR: 3875 (w), 3845 (w), 3807 (w), 3685 (w), 3666 (w), 3492 (w), 3469 (w), 3324 (w), 3253 (w), 3135 (m), 2990 (m), 2602 (m), 2211 (w), 2166 (w), 2151 (w), 2044 (w), 1989 (w), 1972 (w), 1916 (w), 1621 (w), 1528 (s), 1507 (m), 1467 (m), 1435 (m), 1406 (m), 1382 (w), 1250 (s), 1230 (s), 1161 (m), 1136 (s), 1125 (s), 1111 (m), 1084 (s), 1038 (w), 1021 (w), 970 (m), 949 (s), 758 (m), 717 (w), 680 (s), 558 (w), 510 (m), 470 (m), 423 (w), 416 (w).

Guanidinium 5-methyltetrazol-1-olate (6)

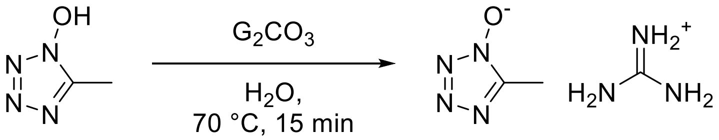

1-Hydroxy-5-methyltetrazole (275 mg, 2.8 mmol, 1 eq.) was dissolved in water (10 mL), guanidinium carbonate (247 mg, 1.4 mmol, 0.5 eq.) was added, the mixture was heated to 80 °C for 15 minutes. The water was evaporated yielding guanidinium 5-methyltetrazol-1-olate as white crystals (365 mg, 2.3 mmol, 92%).

Sensitivities: Bam drop hammer: >40 J, **friction tester**: >360 N

DTA (5 ° min^{-1}): 68 °C(endo), 182 °C (endo), 256 °C (dec).

Elem. Anal. ($C_3H_9N_7O$, 159.15 g mol^{-1}) calcd.: C 22.64, N 61.61, H 5.70%. Found: C 22.83, N 61.62, H 5.56%.

^{1}H NMR (400 MHz, DMSO-*d*$_6$) δ = 7.22 (br s, 6H), 2.15 (s, 3H).

^{13}C NMR (101 MHz, DMSO-*d*$_6$) δ = 158.2, 139.9, 7.9.

IR: 3920 (w), 3711 (w), 3687 (w), 3553 (w), 3520 (w), 3492 (w), 3311 (m), 3272 (m), 3204 (m), 3057 (m), 2826 (m), 2248 (w), 2176 (w), 2151 (w), 2044 (w), 2004 (w), 1988 (w), 1972 (w), 1648 (s), 1590 (m), 1525 (m), 1462 (m), 1410 (m), 1376 (w), 1253

(s), 1242 (s), 1136 (m), 1079 (w), 813 (w), 767 (m), 630 (s), 603 (s), 558 (s), 534 (s), 509 (s), 460 (s), 416 (m), 404 (m).

TATOT 5-methyltetrazol-1-olate (7)

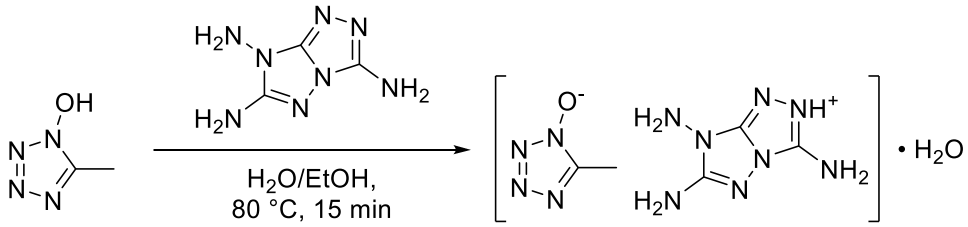

1-Hydroxy-5-methyltetrazole (254 mg, 2.54 mmol, 1 eq.) and TATOT (391 mg, 2.54 mmol, 1 eq.) were suspended in water (3 mL) and EtOH (10 mL). The mixture stirred at 80 °C for 15 minutes. Evaporation of the solvents yielded TATOT 5-methyltetrazol-1-olate monohydrate as off white crystals (505 mg, 2.0 mmol, 78%).

Sensitivities: Bam drop hammer: >40 J, **friction tester**: >360 N

Elem. Anal. ($C_5H_{10}N_{12}O$ • H_2O, 272.23 g mol^{-1}) calcd.: C 22.06, N 61.74, H 4.44%. Found: C 22.13, N 61.70, H 4.76%.

^{1}H NMR (400 MHz, DMSO-d_6) δ = 6.82 (br s, 4H), 5.68 (br s, 2H), 2.23 (s, 3H).

^{13}C NMR (101 MHz, DMSO-d_6) δ = 159.4, 148.2, 143.5, 142.3, 7.4.

IR: 3971 (w), 3924 (w), 3750 (w), 3678 (w), 3473 (w), 3301 (m), 3135 (m), 2233 (w), 2175 (w), 2167 (w), 2161 (w), 2145 (w), 2045 (w), 2005 (w), 1989 (w), 1971 (w), 1939 (w), 1659 (s), 1619 (s), 1579 (m), 1529 (m), 1509 (m), 1427 (m), 1377 (m), 1308 (w), 1288 (w), 1250 (m), 1233 (m), 1158 (m), 1138 (w), 1049 (m), 971 (m), 909 (m), 879 (m), 853 (m), 759 (m), 721 (m), 714 (m), 682 (m), 618 (s), 595 (s), 516 (s), 454 (s), 447 (s), 439 (s), 420 (s).

2. X-Ray Diffraction

Crystal structure data were obtained from an Oxford Xcalibur3 diffractometer with a Spellman generator (voltage 50 kV, current 40 mA) and a Kappa CCD area for data collection using Mo-*K*α radiation (λ = 0.71073 Å) or a Bruker D8 Venture TXS diffractometer equipped with a multilayer monochromator, a Photon 2 detector and a rotation-anode generator (Mo-*Kα* radiation). The data collection was performed using the CRYSTALIS RED software.[S4] The solution of the structure was performed by direct methods and refined by full-matrix leastsquares on F2 (SHELXT)[S5] implemented in the OLEX2[S6] software suite. The non-hydrogen atoms were refined anisotropically and the hydrogen atoms were located and freely refined. The absorption correction was carried out by a SCALE3 ABSPACK multiscan method.[S7] The DIAMOND2 plots shown with thermal ellipsoids at the 50% probability level and hydrogen atoms are shown as small spheres of arbitrary radius. The SADABS program embedded in the Bruker APEX3 software was used for multi-scan absorption corrections in all structures.[S8]

Table S1. Crystallographic data of **2**, **3** and **4**.

	**2**	**3**	**4**
Formula	$C_2H_4N_4O$	$C_2H_7N_8O$	$C_2H_7N_5O_2$
FW [g mol^{-1}]	100.09	117.13	133.13
Crystal system	orthorhombic	orthorhombic	triclinic
Space group	Pna21 (No. 33)	Pbca (No. 61)	P-1 (No. 2)
Color / Habit	colourless block	colourless block	colourless block
Size [mm]	0.06 x 0.14 x 0.16	0.50 x 0.20 x 0.20	0.06 x 0.08 x 0.13
a [Å]	9.6928(4)	13.1916(8)	7.3821(5)
b [Å]	4.0112(2)	7.5481(4)	8.2511(6)
c [Å]	11.4614(5)	21.4023(10)	10.2099(8)
α [°]	90	90	84.751(2)
β [°]	90	90	75.067(3)
γ [°]	90	90	82.060(2)
V [Å^3]	445.62(3)	2131.1(2)	594.11(8)
Z	4	16	4
$\rho_{calc.}$ [g cm^{-3}]	1.492	1.460	1.488
μ [mm^{-1}]	0.122	0.119	0.128
F(000)	208	992	280
$\lambda_{MoK\alpha}$ [Å]	0.71073	0.71073	0.71073
T [K]	173	173	173
θ Min-Max [°]	4.2, 30.6	2.5, 30.5	2.5, 27.5
Dataset	-13: 13 ; -5: 5 ; -16: 16	-14: 18 ; -9: 10 ; -30: 26	-9: 9; -10: 10; -13: 13
Reflections collected	11216	7821	10329
Independent refl.	1355	3260	2742
R_{int}	0.032	0.052	0.041
Observed reflections	1284	2101	2380
Parameters	69	201	219
R_1 (obs)[a]	0.0376	0.0546	0.0414
wR_2 (all data)[b]	0.1049	0.1209	0.1124
S [c]	1.10	1.02	1.07
Resd. dens [e Å^{-3}]	-0.17, 0.24	-0.25, 0.28	-0.27, 0.30
Device type	Xcalibur Sapphire3	Xcalibur Sapphire3	Bruker D8 Venture
Solution	SIR-92	SIR-92	SIR-92
Refinement	SHELXL-2013	SHELXL-2013	SHELXL-2013
Absorption correction	multi-scan	multi-scan	multi-scan
CCDC	2453219	2453217	2453215

[a]$R_1 = \Sigma||F_0|-|F_c||/\Sigma|F_0|$; [b]$wR_2 = [\Sigma[w(F_0^2-F_c^2)^2]/\Sigma[w(F_0)^2]]^{1/2}$; $w = [\sigma c^2(F_0^2)+(xP)^2+yP]^{-1}$ and $P=(F_0^2+2F_c^2)/3$; [c]$S = \{\Sigma[w(F_o^2-F_c^2)^2]/(n-p)\}^{1/2}$ (n = number of reflections; p = total number of parameters).

Table S2. Crystallographic data of **5**, **6** and **7**.

	**5**	**6**	**7**
Formula	$C_2H_8N_6O$	$C_3H_9N_7O$	$C_5H_{10}N_{12}O\cdot H_2O$
FW [g mol^{-1}]	132.14	159.17	272.27
Crystal system	orthorhombic	monoclinic	triclinic
Space group	Pbca (No. 61)	C2/c (No. 15)	P-1 (No. 2)
Color / Habit	colourless plate	colourless rod	colourless block
Size [mm]	0.01 x 0.25 x 0.50	0.13 x 0.16 x 0.28	0.15 x 0.20 x 0.30
a [Å]	14.632(2)	11.9689(8)	6.4887(7)
b [Å]	6.9287(11)	11.2025(7)	6.9104(8)
c [Å]	24.028(5)	11.6752(6)	13.2806(14)
α [°]	90	90	96.990(9)
β [°]	90	104.309(6)	94.897(9)
γ [°]	90	90	104.023(10)
V [Å^3]	2436.0(7)	1516.87(16)	569.36(11)
Z	16	8	2
$\rho_{calc.}$ [g cm^{-3}]	1.441	1.394	1.588
μ [mm^{-1}]	0.117	0.111	0.128
F(000)	1120	672	284
$\lambda_{MoK\alpha}$ [Å]	0.71073	0.71073	0.71073
T [K]	123	173	123
θ Min-Max [°]	2.2, 26.4	2.8, 29.0	3.1, 26.4
Dataset	-18: 18 ; -8: 7 ; -30: 29	-14: 13 ; -15: 9 ; -14: 14	-8: 8 ; -7: 8 ; -16: 16
Reflections collected	18005	3305	4755
Independent refl.	2491	1726	2334
R_{int}	0.076	0.022	0.026
Observed reflections	1805	1381	1902
Parameters	205	136	220
R_1 (obs)[a]	0.0436	0.0385	0.0385
wR_2 (all data)[b]	0.0997	0.0961	0.0919
S [c]	1.04	1.04	1.06
Resd. dens [e Å^{-3}]	-0.19, 0.25	-0.19, 0.16	-0.28, 0.21
Device type	Xcalibur Sapphire3	Xcalibur Sapphire3	Xcalibur Sapphire3
Solution	SIR-92	SIR-92	SIR-92
Refinement	SHELXL-2013	SHELXL-2013	SHELXL-2013
Absorption correction	multi-scan	multi-scan	multi-scan
CCDC	2453218	2453216	2453214

[a]$R_1 = \Sigma||F_0|-|F_c||/\Sigma|F_0|$; [b]$wR_2 = [\Sigma[w(F_0^2-F_c^2)^2]/\Sigma[w(F_0)^2]]^{1/2}$; $w = [\sigma c^2(F_0^2)+(xP)^2+yP]^{-1}$ and $P=(F_0^2+2F_c^2)/3$; [c]$S = \{\Sigma[w(F_o^2-F_c^2)^2]/(n-p)\}^{1/2}$ (n = number of reflections; p = total number of parameters).

3. Computation

All quantum chemical calculations were carried out using the Gaussian G09 program package.[S9] The enthalpies (H) and free energies (G) were calculated using the complete basis set (CBS) method of Petersson and co-workers in order to obtain very accurate energies. The CBS models are using the known asymptotic convergence of pair natural orbital expressions to extrapolate from calculations using a finite basis set to the estimated CBS limit. CBS-4 starts with an HF/3-21G(d) geometry optimization; the zero-point energy is computed at the same level. It then uses a large basis set SCF calculation as a base energy, and an MP2/6- 31+G calculation with a CBS extrapolation to correct the energy through second order. A MP4(SDQ)/6-31+ (d,p) calculation is used to approximate higher order contributions. In this study, we applied the modified CBS-4M.

Heats of formation of the synthesized ionic compounds were calculated using the atomization method (equation E1) using room temperature CBS-4M enthalpies, which are summarized in Table 4.[S10, S11]

$$\Delta_f H^\circ_{(g,\, M,\, 298)} = H_{(Molecule,\, 298)} - \sum H^\circ_{(Atoms,\, 298)} + \sum \Delta_f H^\circ_{(Atoms,\, 298)} \qquad (E1)$$

Table S3. CBS-4M electronic enthalpies for atoms C, H, N and O and their literature values for atomic $\Delta H^\circ{}_f{}^{298}$ / kJ mol^{-1}.

	$-H^{298}$ [a.u.]	NIST[S12]
H	0.500991	218.2
C	37.786156	717.2
N	54.522462	473.1
O	74.991202	249.5

For neutral compounds the sublimation enthalpy, which is needed to convert the gas phase enthalpy of formation to the solid state one, was calculated by the *Trouton* rule.[S13] For ionic compounds, the lattice energy (U_L) and lattice enthalpy (ΔH_L) were calculated from the corresponding X-ray molecular volumes according to the equations provided by *Jenkins* and *Glasser*.[S14] With the calculated lattice enthalpy the gas-phase enthalpy of formation was converted into the solid state (standard conditions) enthalpy of formation. These molar standard enthalpies of formation (ΔH_m) were used to calculate the molar solid-state energies of formation (ΔU_m) according to equation E2.

$$\Delta U_m = \Delta H_m - \Delta n\, RT \qquad \text{(E2)}$$

(Δn being the change of moles of gaseous components)

The calculation results are summarized in Table 4.

Table S4. Calculation results.

	$-H^{298}$ [a] [a.u.]	$\Delta_f H°_{(g)}$(M) [kJ mol^{-1}] [b]	V_M [Å^3] [c]	ΔU_L; ΔH_L [d] [kJ mol^{-1}]	$\Delta_f H°$(s) [e] [kJ mol^{-1}]	$-\Delta n$ [f]	$\Delta_f U$(s) [g] [kJ kg^{-1}]
**A^-**	371.724406	110.3					
**NH_4^+**	56.796608	635.3					
**NH_3OH^+**	131.863229	686.4					
**$N_2H_5^+$**	112.030523	773.4					
**G^+**	205.453192	571.2					
**2**	372.245833	274.6			230.5	4.5	2414.8
**3**	-	745.3	133	563.2; 568.1	177.5	6.5	1653.0
**4**	-	796.7	149	546.8; 551.7	262.3	7.0	1970.7
**5**	-	883.7	152	543.1, 548.1	335.6	7.5	2680.8
**6**	-	681.5	190	512.2; 517.1	164.4	8.5	1165.1

[a] CBS-4M electronic enthalpy; [b] gas phase enthalpy of formation; [c] molecular volumes taken from X-ray structures and corrected to room temperature; [d] lattice energy and enthalpy (calculated using Jenkins and Glasser equations); [e] standard solid-state enthalpy of formation; [f] Δn being the change of moles of gaseous components when formed; [g] solid state energy of formation.

4. NMR Spectroscopy

^{1}H NMR of 1-hydroxy-5-methyltetrazole (2)

DMSO-d_6, 400 MHz

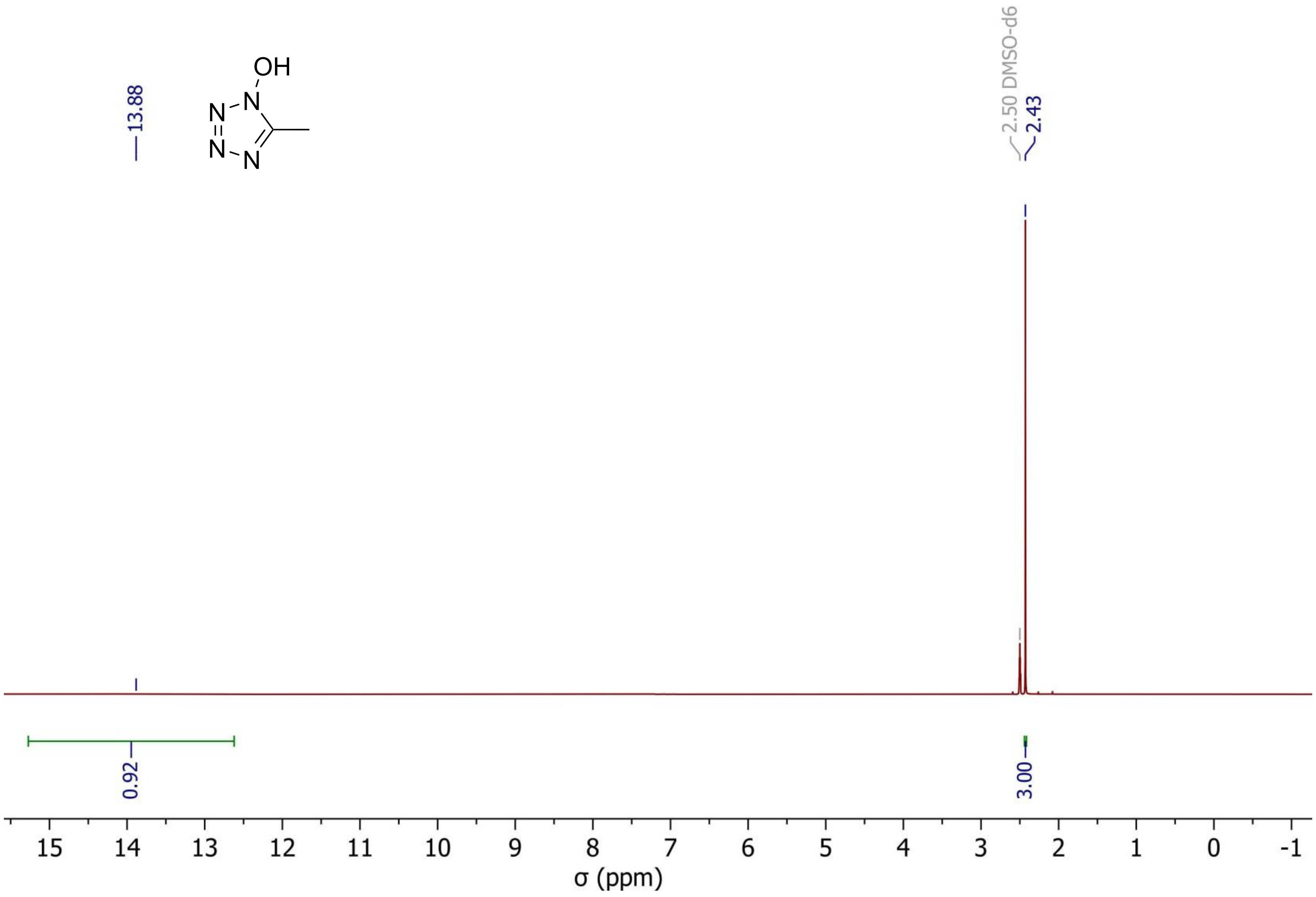

^{13}C NMR of 1-hydroxy-5-methyltetrazole (2)

DMSO-d_6, 101 MHz

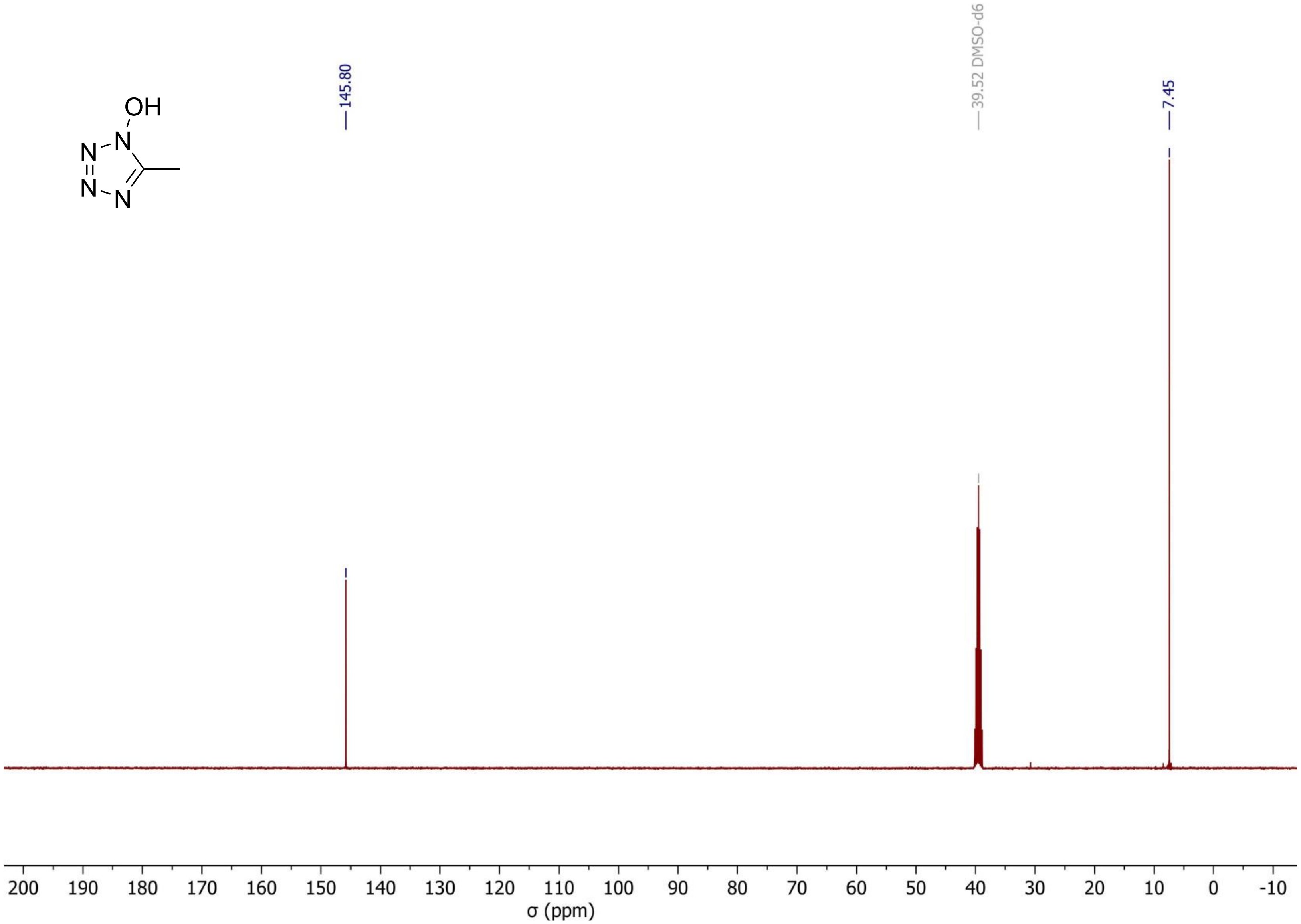

^{1}H NMR of ammonium 1-hydroxy-5-methyltetrazolate (3)

DMSO-d_6, 400 MHz

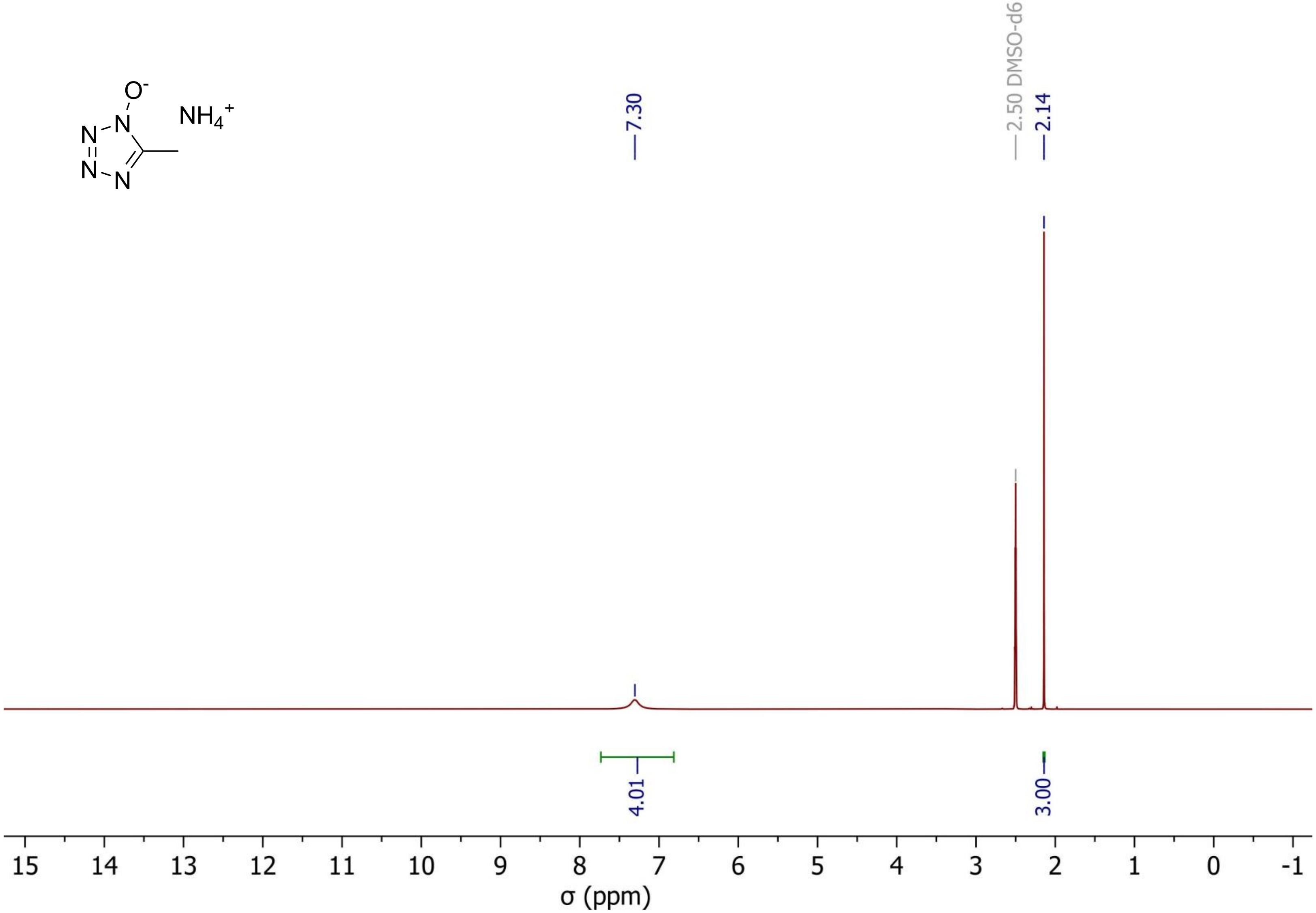

^{13}C NMR of ammonium 1-hydroxy-5-methyltetrazolate (3)

DMSO-d_6, 101 MHz

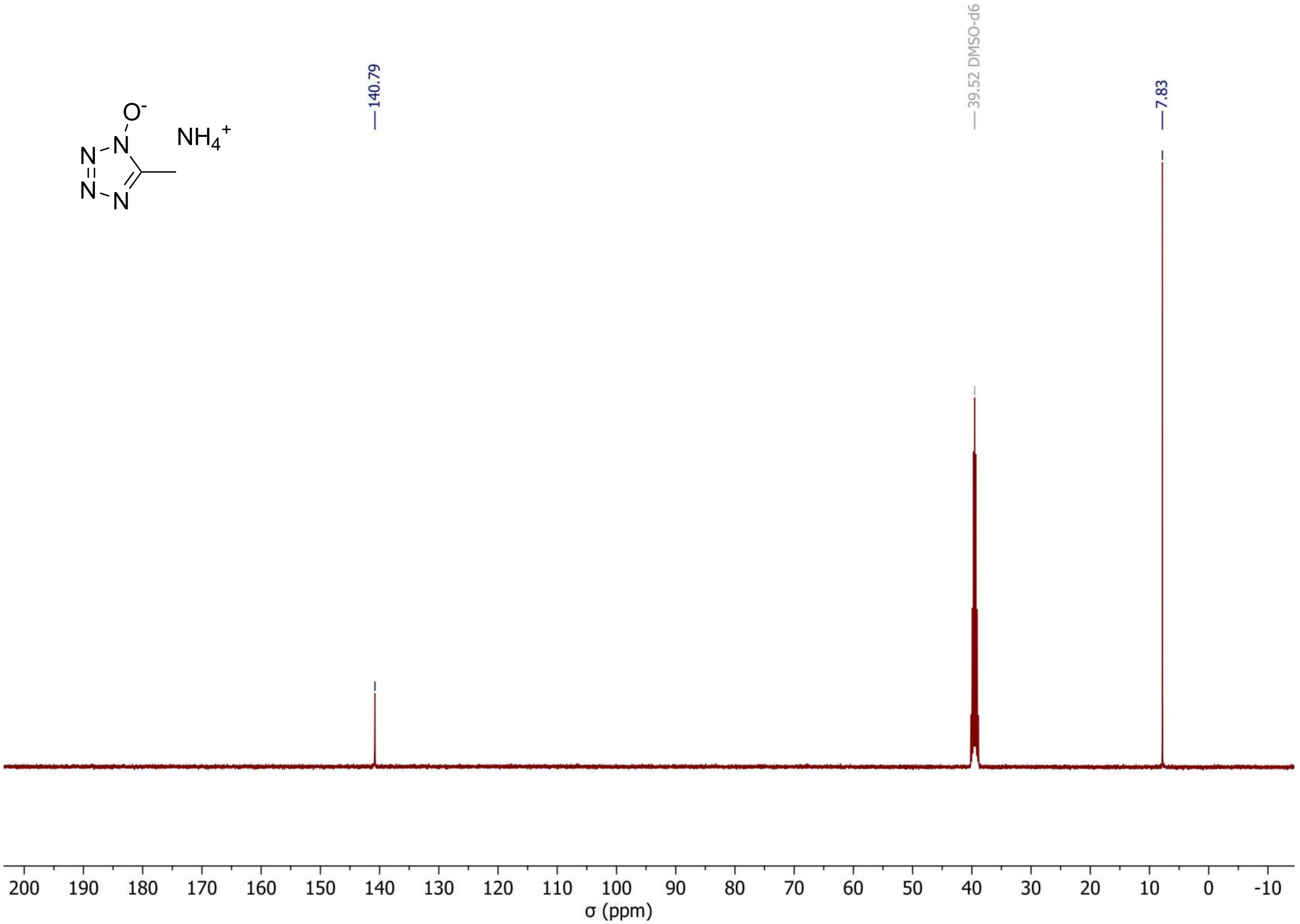

^{1}H NMR of hydroxylammonium 1-hydroxy-5-methyltetrazolate (4)

DMSO-d_6, 400 MHz

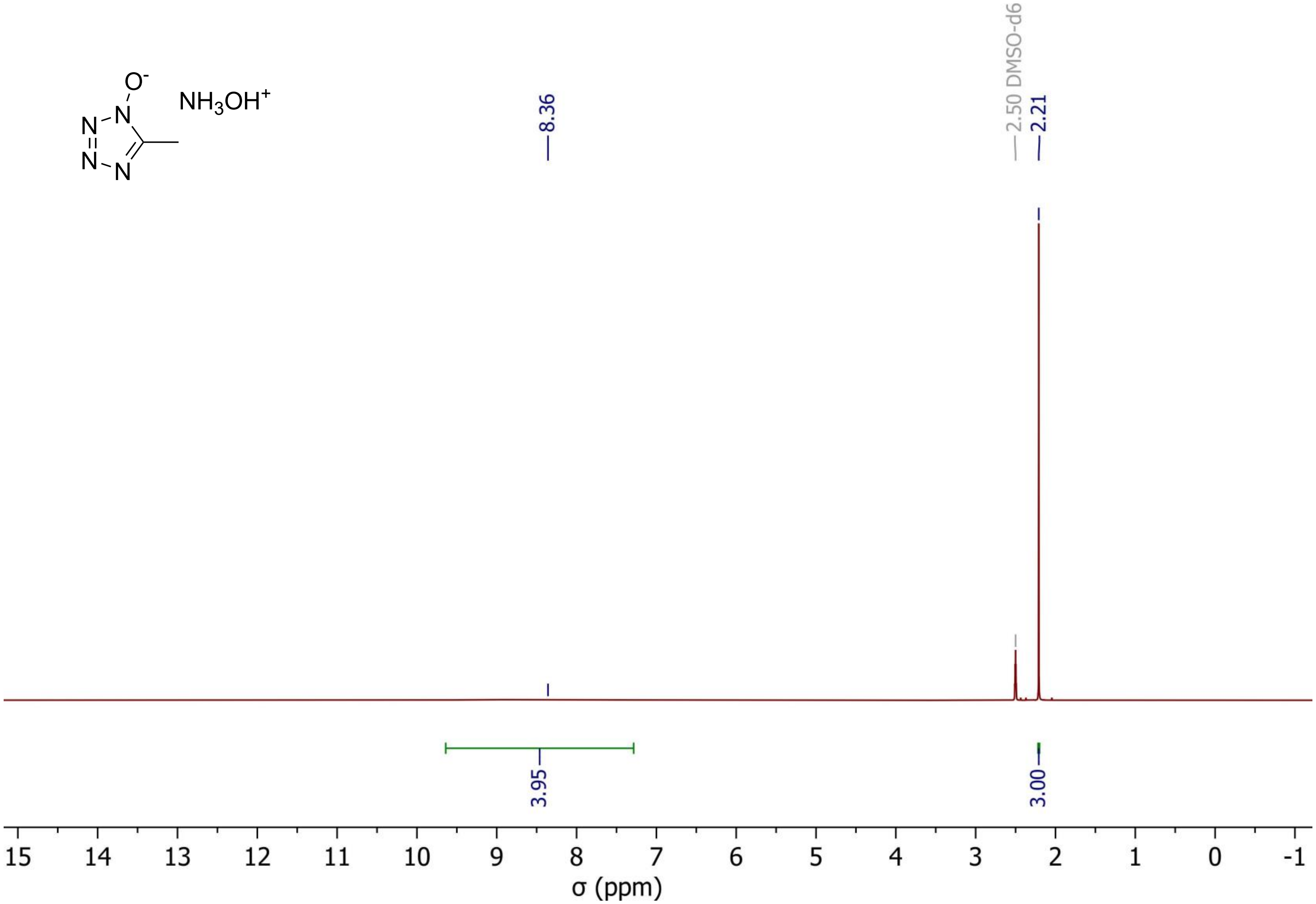

^{13}C NMR of hydroxylammonium 1-hydroxy-5-methyltetrazolate (4)

DMSO-d_6, 101 MHz

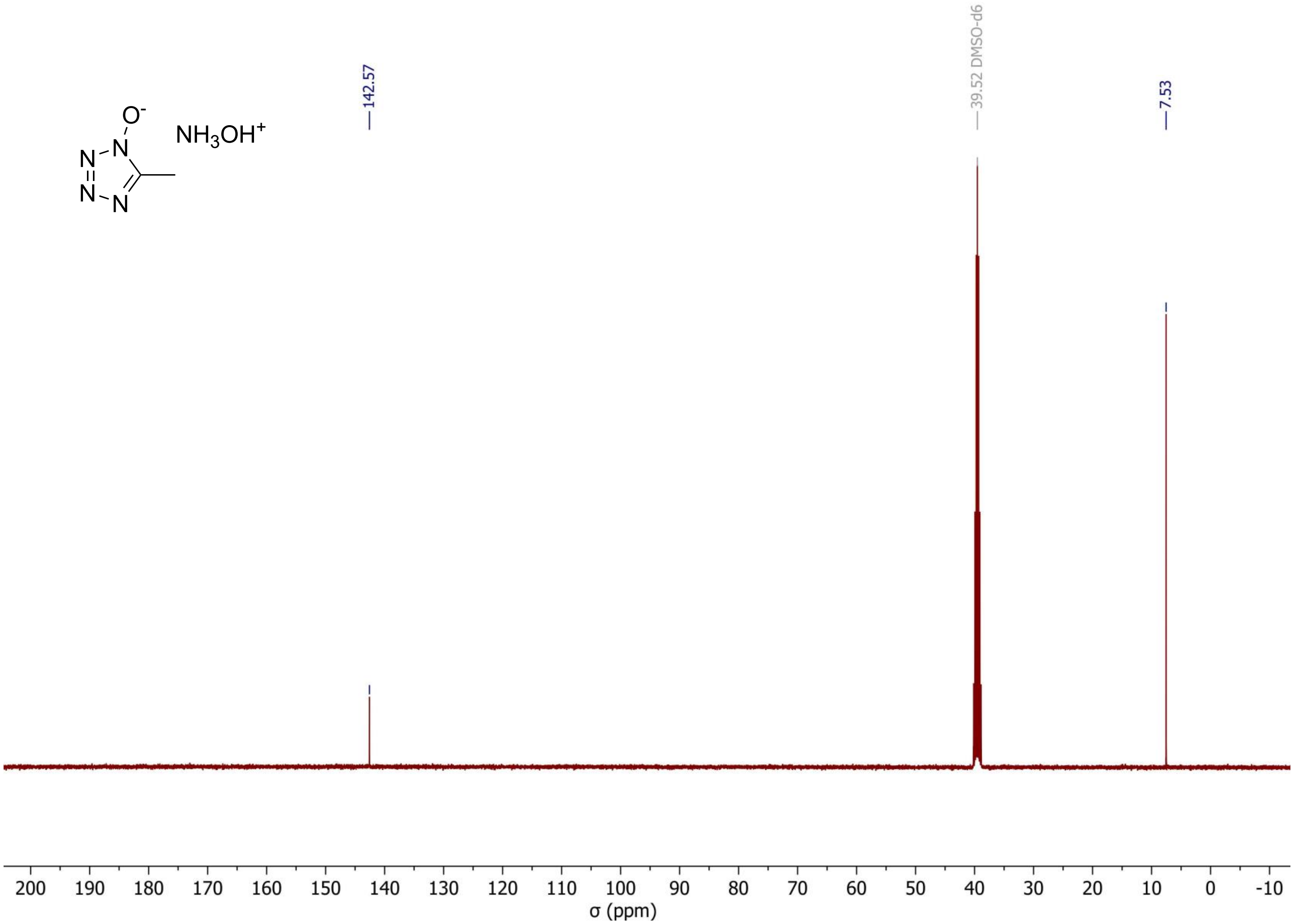

^{1}H NMR of hydrazinium 1-hydroxy-5-methyltetrazolate (5)

DMSO-d_6, 400 MHz

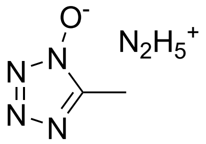

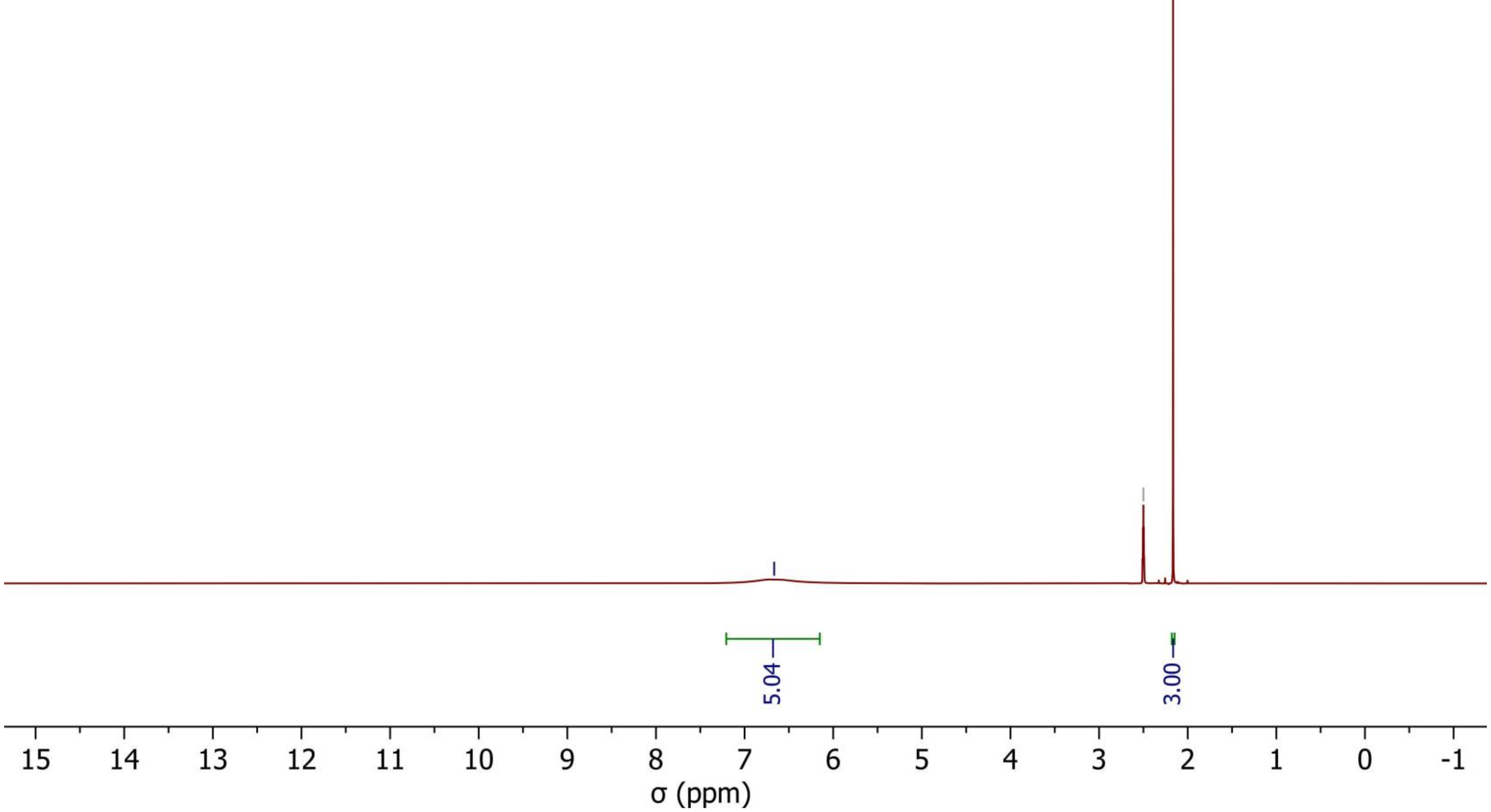

^{13}C NMR of hydrazinium 1-hydroxy-5-methyltetrazolate (5)

DMSO-d_6, 101 MHz

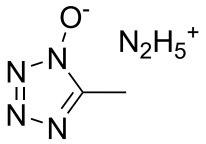

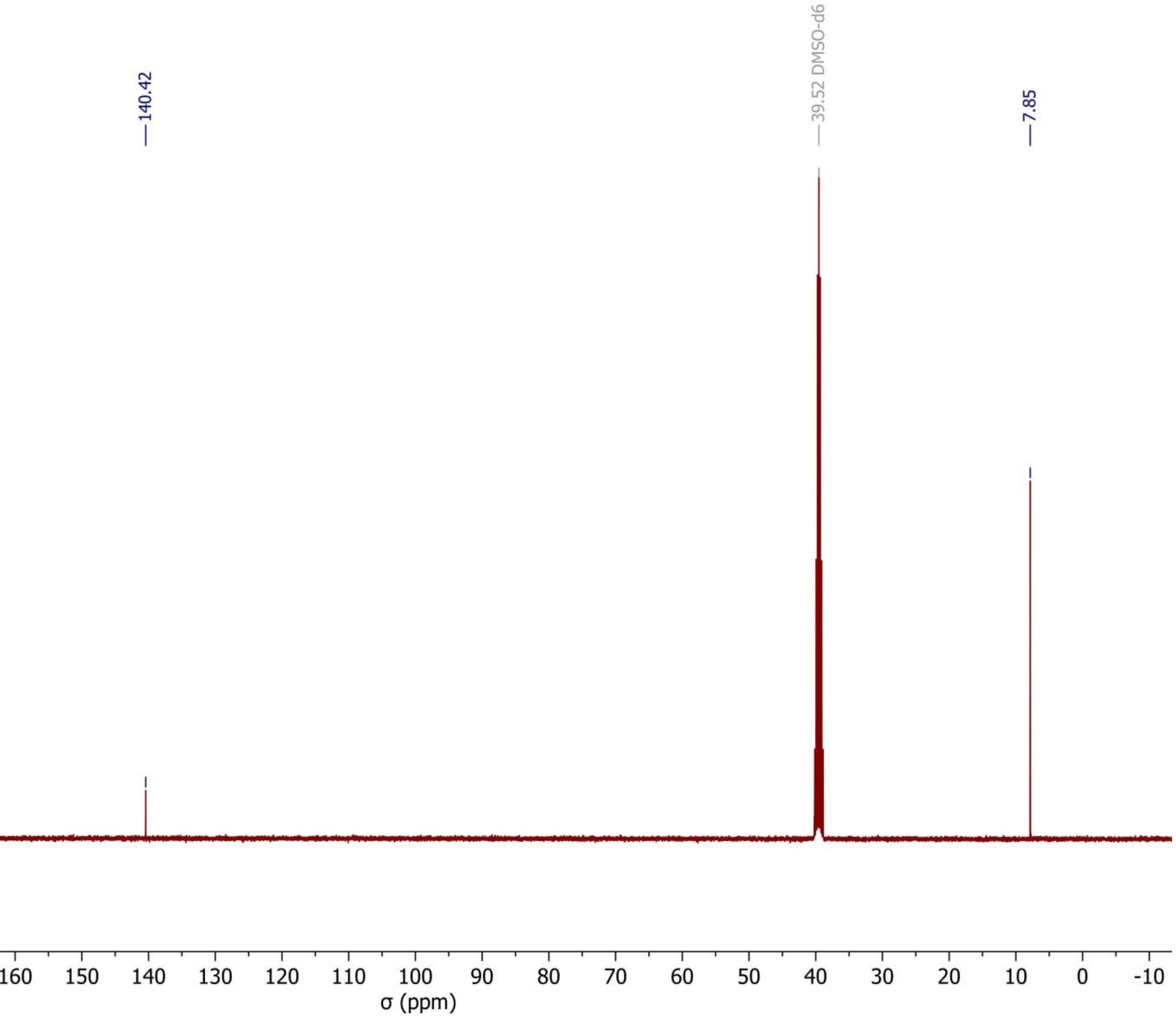

^{1}H NMR of guanidinium 1-hydroxy-5-methyltetrazolate (6)

DMSO-d_6, 400 MHz

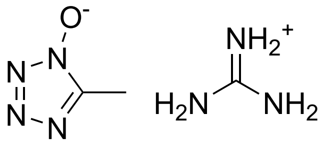

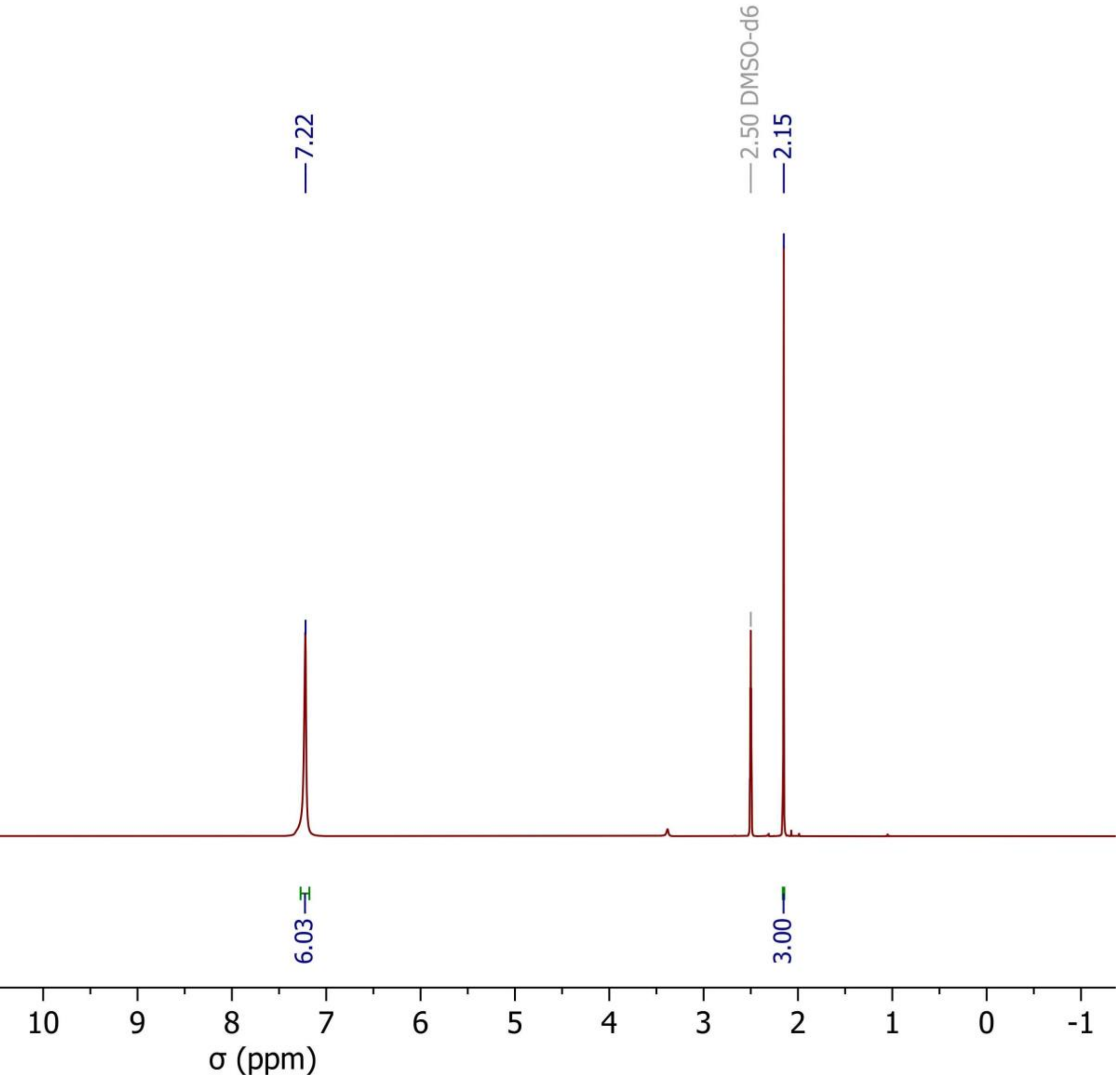

^{13}C NMR of guanidinium 1-hydroxy-5-methyltetrazolate (6)

DMSO-d_6, 101 MHz

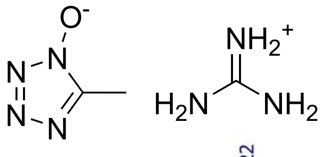

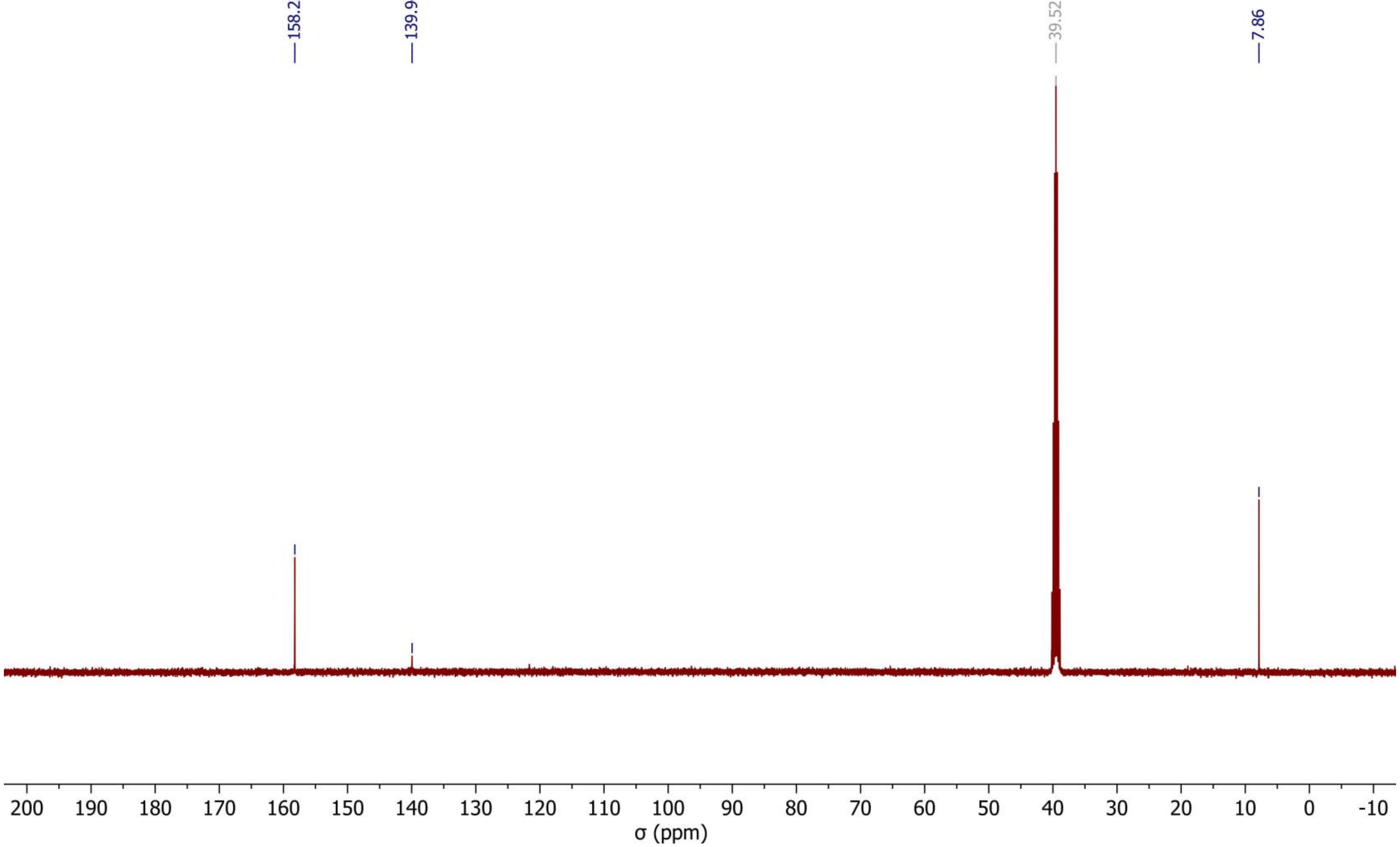

^{1}H NMR of Htatot 1-hydroxy-5-methyltetrazolate (7)

DMSO-d_6, 400 MHz

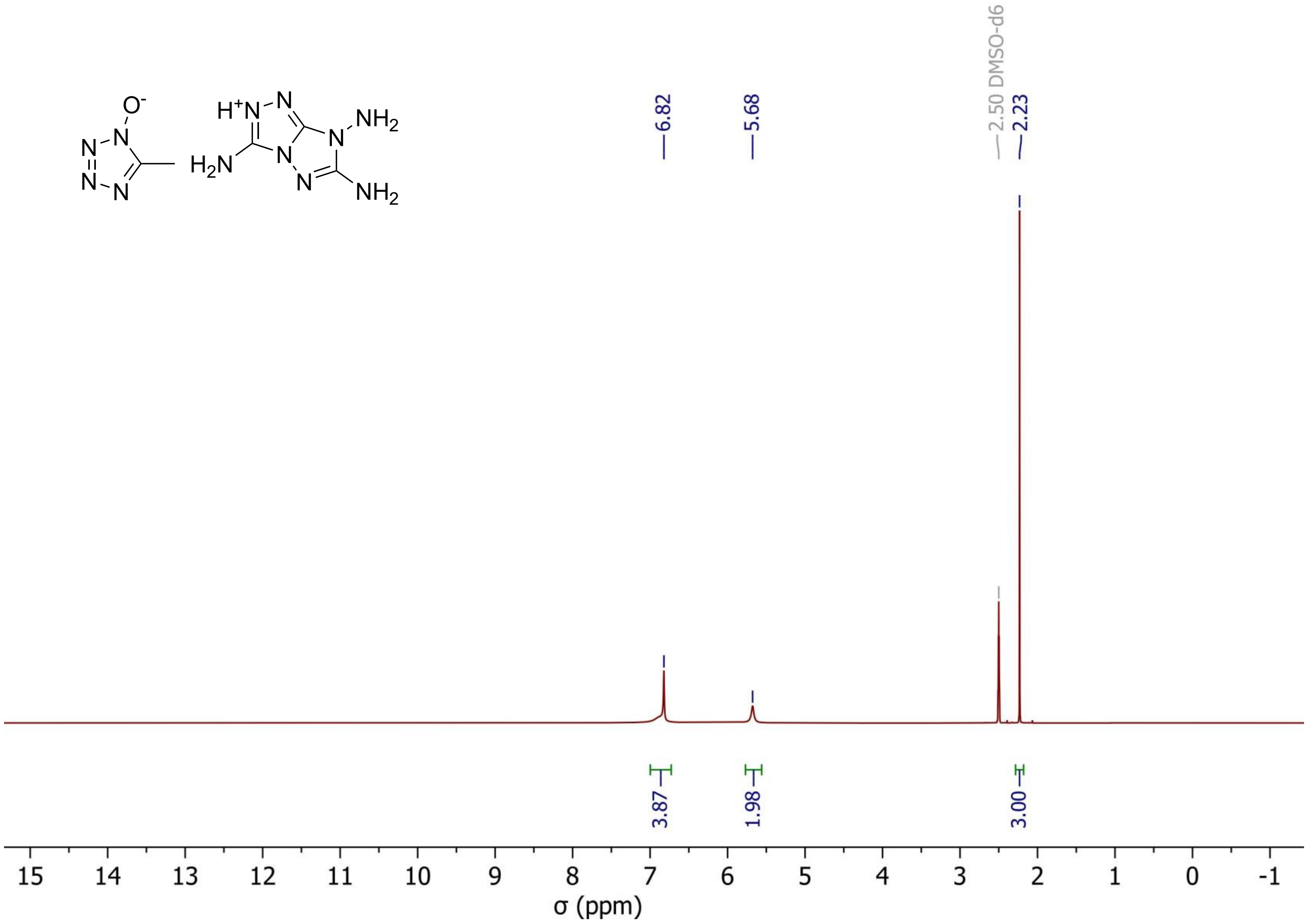

^{13}C NMR of Htatot 1-hydroxy-5-methyltetrazolate (7)

DMSO-d_6, 101 MHz

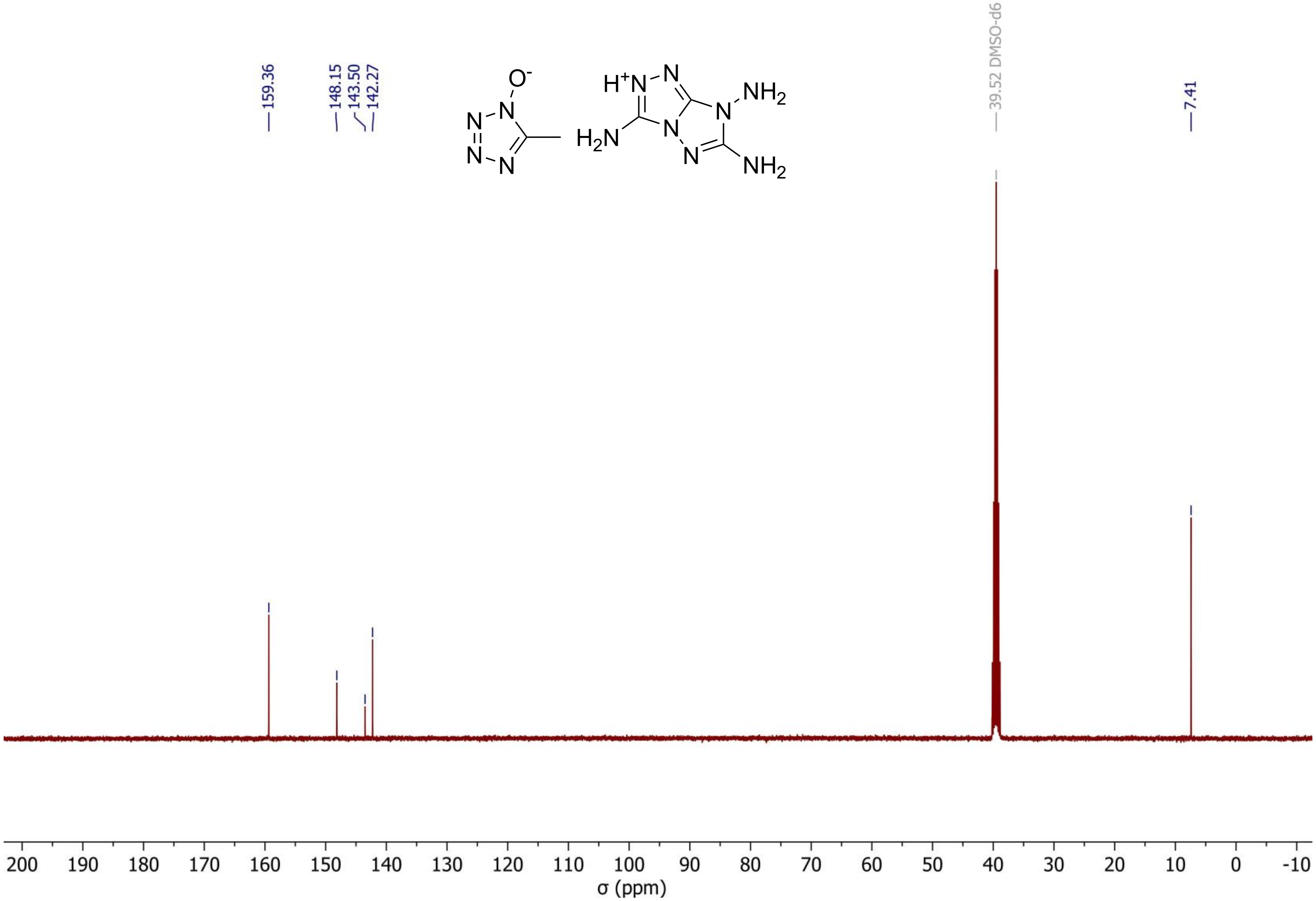

5. Toxicity

Predictions were performed using the ProTox-3.0 web-based platform, with SMILES format as the input. Acute toxicity, carcinogenicity, mutagenicity and ecotoxicity were predicted. [S15, S16]

1-Hydroxy-5-methyltetrazole (2):

Oral toxicity prediction results for input compound

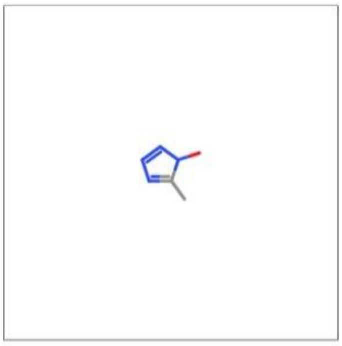

Predicted LD50: 890mg/kg

Predicted Toxicity Class: 4

1 2 3 4 5 6

Average similarity: 31.42%

Prediction accuracy: 23%

20% 40% 60% 80%

Name	CC1=NN=NN1O
Molweight	100.08
Number of hydrogen bond acceptors	4
Number of hydrogen bond donors	1
Number of atoms	7
Number of bonds	7
Number of rotable bonds	0
Molecular refractivity	20.33
Topological Polar Surface Area	63.83
octanol/water partition coefficient(logP)	-0.78

Toxicity Model Report

Copy Excel CSV PDF

Classification	Target	Shorthand	Prediction	Probability
Toxicity end points	Carcinogenicity	carcino	**Active**	0.59
Toxicity end points	Mutagenicity	mutagen	Inactive	0.62
Toxicity end points	Ecotoxicity	eco	Inactive	0.59

Toxicity targets

Possible binding to toxicity targets is shown below. For more information on the targets, please click on the individual abbreviations.

no binding probable binding

AA2AR	ADRB2	ANDR	AOFA	CRFR1	DRD3	ESR1	ESR2	GCR	HRH1	NR1I2	OPRK	OPRM	PDE4D	PGH1	PRGR

Ammonium 1-hydroxy-5-methyltetrazolate (3):

Oral toxicity prediction results for input compound

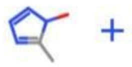

Predicted LD50: 890mg/kg

Predicted Toxicity Class: 4

1 2 3 4 5 6

Average similarity: 31.42%

Prediction accuracy: 23%

20% 40% 60% 80%

Name	[H][N+]([H])([H])[H].CC1=NN=NN1[O-]
Molweight	117.11
Number of hydrogen bond acceptors	3
Number of hydrogen bond donors	1
Number of atoms	8
Number of bonds	7
Number of rotable bonds	0
Molecular refractivity	27.53
Topological Polar Surface Area	66.66
octanol/water partition coefficient(logP)	-0.3

Toxicity Model Report

Copy Excel CSV PDF

Classification	Target	Shorthand	Prediction	Probability
Toxicity end points	Carcinogenicity	carcino	**Active**	0.62
Toxicity end points	Mutagenicity	mutagen	Inactive	0.52
Toxicity end points	Ecotoxicity	eco	Inactive	0.55

Toxicity targets

Possible binding to toxicity targets is shown below. For more information on the targets, please click on the individual abbreviations.

no binding probable binding

AA2AR	ADRB2	ANDR	AOFA	CRFR1	DRD3	ESR1	ESR2	GCR	HRH1	NR1I2	OPRK	OPRM	PDE4D	PGH1	PRGR

Hydroxylammonium 1-hydroxy-5-methyltetrazolate (4):

Oral toxicity prediction results for input compound

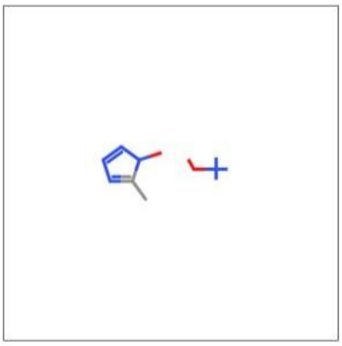

Predicted LD50: 890mg/kg

Predicted Toxicity Class: 4

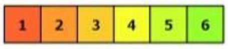

Average similarity: 31.42%

Prediction accuracy: 23%

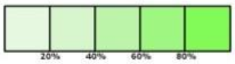

Name	CC1=NN=NN1[O-].[H][N+]([H])([H])O[H]
Molweight	133.11
Number of hydrogen bond acceptors	4
Number of hydrogen bond donors	2
Number of atoms	9
Number of bonds	8
Number of rotable bonds	0
Molecular refractivity	28.36
Topological Polar Surface Area	114.53
octanol/water partition coefficient(logP)	-2.06

Toxicity Model Report

Copy Excel CSV PDF

Classification	Target	Shorthand	Prediction	Probability
Toxicity end points	Carcinogenicity	carcino	**Active**	0.58
Toxicity end points	Mutagenicity	mutagen	Inactive	0.59
Toxicity end points	Ecotoxicity	eco	Inactive	0.70

Toxicity targets

Possible binding to toxicity targets is shown below. For more information on the targets, please click on the individual abbreviations.

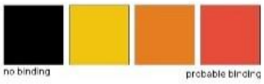

AA2AR	ADRB2	ANDR	AOFA	CRFR1	DRD3	ESR1	ESR2	GCR	HRH1	NR1I2	OPRK	OPRM	PDE4D	PGH1	PRGR

Hydrazinium 1-hydroxy-5-methyltetrazolate (5):

Oral toxicity prediction results for input compound

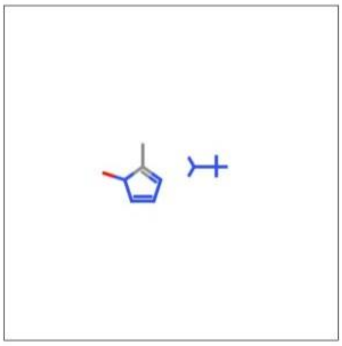

Predicted LD50: 890mg/kg

Predicted Toxicity Class: 4

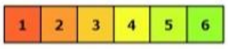

Average similarity: 30.79%

Prediction accuracy: 23%

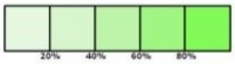

Name	CC1=NN=NN1[O-].[H][N+]([H])([H])N([H])[H]
Molweight	132.13
Number of hydrogen bond acceptors	4
Number of hydrogen bond donors	2
Number of atoms	9
Number of bonds	8
Number of rotable bonds	0
Molecular refractivity	30.33
Topological Polar Surface Area	120.32
octanol/water partition coefficient(logP)	-1.87

Toxicity Model Report

Copy Excel CSV PDF

Classification	Target	Shorthand	Prediction	Probability
Toxicity end points	Carcinogenicity	carcino	**Active**	0.63
Toxicity end points	Mutagenicity	mutagen	Inactive	0.54
Toxicity end points	Ecotoxicity	eco	Inactive	0.71

Toxicity targets

Possible binding to toxicity targets is shown below. For more information on the targets, please click on the individual abbreviations.

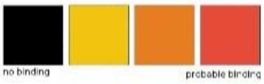

AA2AR	ADRB2	ANDR	AOFA	CRFR1	DRD3	ESR1	ESR2	GCR	HRH1	NR1I2	OPRK	OPRM	PDE4D	PGH1	PRGR

Guanidinium 1-hydroxy-5-methyltetrazolate (6):

Oral toxicity prediction results for input compound

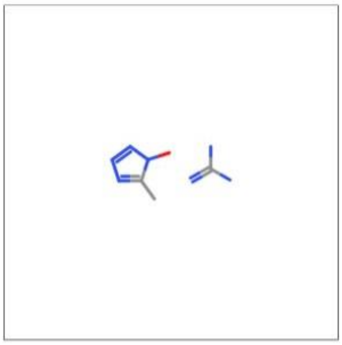

Predicted LD50: 3150mg/kg

Predicted Toxicity Class: 5

1 2 3 4 5 6

Average similarity: 30.21%

Prediction accuracy: 23%

20% 40% 60% 80%

Name	NC(N)=[NH2+].CC1=NN=
Molweight	159.15
Number of hydrogen bond acceptors	5
Number of hydrogen bond donors	3
Number of atoms	11
Number of bonds	10
Number of rotable bonds	0
Molecular refractivity	38.14
Topological Polar Surface Area	144.29
octanol/water partition coefficient(logP)	-2.25

Toxicity Model Report

Copy Excel CSV PDF

Classification	Target	Shorthand	Prediction	Probability
Toxicity end points	Carcinogenicity	carcino	**Active**	0.69
Toxicity end points	Mutagenicity	mutagen	**Active**	0.61
Toxicity end points	Ecotoxicity	eco	Inactive	0.62

Toxicity targets

Possible binding to toxicity targets is shown below. For more information on the targets, please click on the individual abbreviations.

no binding probable binding

AA2AR	ADRB2	ANDR	AOFA	CRFR1	DRD3	ESR1	ESR2	GCR	HRH1	NR1I2	OPRK	OPRM	PDE4D	PGH1	PRGR

TNT (2,4,6-trinitrotoluene):

Oral toxicity prediction results for input compound

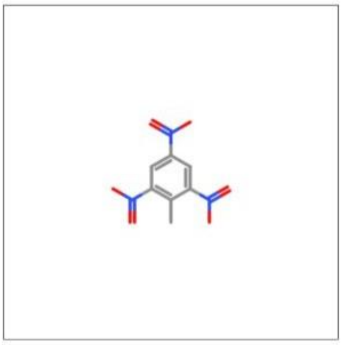

Predicted LD50: 607mg/kg

Predicted Toxicity Class: 3

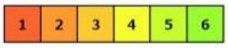

Average similarity: 71.11%

Prediction accuracy: 69.26%

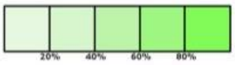

Name	CC1=C([N+]([O-])=O)C=C([N+]([O-])=O)C=C1[N+]([O-])=O
Molweight	227.13
Number of hydrogen bond acceptors	3
Number of hydrogen bond donors	0
Number of atoms	16
Number of bonds	16
Number of rotable bonds	3
Molecular refractivity	57.87
Topological Polar Surface Area	137.46
octanol/water partition coefficient(logP)	3.29

Toxicity Model Report

Copy Excel CSV PDF

Classification	Target	Shorthand	Prediction	Probability
Toxicity end points	Carcinogenicity	carcino	Inactive	0.55
Toxicity end points	Mutagenicity	mutagen	**Active**	0.99
Toxicity end points	Ecotoxicity	eco	**Active**	0.77

Toxicity targets

Possible binding to toxicity targets is shown below. For more information on the targets, please click on the individual abbreviations.

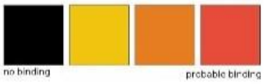

AA2AR	ADRB2	ANDR	AOFA	CRFR1	DRD3	ESR1	ESR2	GCR	HRH1	NR1I2	OPRK	OPRM	PDE4D	PGH1	PRGR

RDX (1,3,5-trinitro-1,3,5-triazinane):

Oral toxicity prediction results for input compound

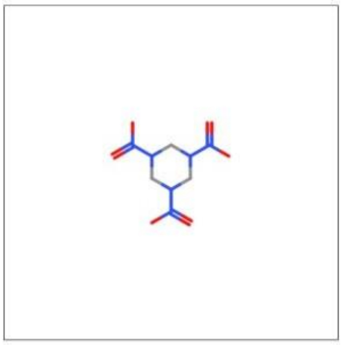

Predicted LD50: 100mg/kg

Predicted Toxicity Class: 3

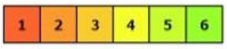

Average similarity: 69.24%

Prediction accuracy: 68.07%

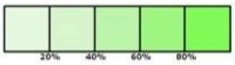

Name	O=[N+](N1CN([N+]([O-])=O)CN([N+]([O-])=O)C1)[O-]
Molweight	222.12
Number of hydrogen bond acceptors	6
Number of hydrogen bond donors	0
Number of atoms	15
Number of bonds	15
Number of rotable bonds	3
Molecular refractivity	59.17
Topological Polar Surface Area	147.18
octanol/water partition coefficient(logP)	-0.26

Toxicity Model Report

Copy Excel CSV PDF

Classification	Target	Shorthand	Prediction	Probability
Toxicity end points	Carcinogenicity	carcino	**Active**	0.86
Toxicity end points	Mutagenicity	mutagen	**Active**	0.89
Toxicity end points	Ecotoxicity	eco	**Active**	0.50

Toxicity targets

Possible binding to toxicity targets is shown below. For more information on the targets, please click on the individual abbreviations.

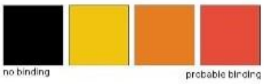

AA2AR	ADRB2	ANDR	AOFA	CRFR1	DRD3	ESR1	ESR2	GCR	HRH1	NR1I2	OPRK	OPRM	PDE4D	PGH1	PRGR

HMX (1,3,5,7-tetranitro-1,3,5,7-tetrazocane):

Oral toxicity prediction results for input compound

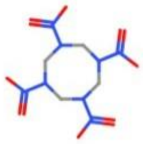

Predicted LD50: 186mg/kg

Predicted Toxicity Class: 3

1	2	3	4	5	6

Average similarity: 64.59%

Prediction accuracy: 68.07%

20% 40% 60% 80%

Name	O=[N+](N1CN([N+]([O-])=O)CN([N+]([O-])=O)CN([N+]([O-])=O)C1)[O-]
Molweight	296.16
Number of hydrogen bond acceptors	8
Number of hydrogen bond donors	0
Number of atoms	20
Number of bonds	20
Number of rotable bonds	4
Molecular refractivity	78.89
Topological Polar Surface Area	196.24
octanol/water partition coefficient(logP)	-0.35

Toxicity Model Report

Copy Excel CSV PDF

Classification	Target	Shorthand	Prediction	Probability
Toxicity end points	Carcinogenicity	carcino	**Active**	0.87
Toxicity end points	Mutagenicity	mutagen	**Active**	0.87
Toxicity end points	Ecotoxicity	eco	**Active**	0.53

Toxicity targets

Possible binding to toxicity targets is shown below. For more information on the targets, please click on the individual abbreviations.

no binding probable binding

AA2AR	ADRB2	ANDR	AOFA	CRFR1	DRD3	ESR1	ESR2	GCR	HRH1	NR1I2	OPRK	OPRM	PDE4D	PGH1	PRGR

6. References

[S1] a) Reichel & Partner GmbH, http://www.reichel-partner.de; b) Test methods according to the UN Recommendations on the Transport of Dangerous Goods, Manual of Test and Criteria, fourth revised edition, United Nations Publication, New York and Geneva, 2003, ISBN 92–1-139087–7, Sales No. E.03.VIII.2; 13.4.2 Test 3 a (ii) BAM Fallhammer.

[S2] Z. P. Demko, K. B. Sharpless *J. Org. Chem.* **2001**, *66*, 24, 7945–7950.

[S3] L. Bauer, M. Benz, T. M. Klapötke, C. Pignot, J. Stierstorfer *Mater. Adv.* **2022**, *3*, 3945–3951

[S4] *CrysAlisPro*, Oxford Diffraction Ltd. *version 171.33.41*, **2009**.

[S5] Sheldrick, G.M., *Acta Cryst.* **2015**, *A71*, 3–8.

[S6] Dolomanov, O. V.; Bourhis, L. J.; Gildea, R. J.; Howard, J. A. K.; Puschmann, H., OLEX2: A complete structure solution, refinement and analysis program, *J. Appl. Cryst.* **2009**, *42*, 339-341.

[S7] *SCALE3 ABSPACK – An Oxford Diffraction program* (1.0.4, gui: 1.0.3), Oxford Diffraction Ltd., **2005**.

[S8] *APEX3*. Bruker AXS Inc., Madison, Wisconsin, USA.

[S9] M. J. Frisch, G. W. Trucks, H. B. Schlegel, G. E. Scuseria, M. A. Robb, J. R. Cheeseman, G. Scalmani, V. Barone, B. Mennucci, G. A. Petersson, H. Nakatsuji, M. Caricato, X. Li, H.P. Hratchian, A. F. Izmaylov, J. Bloino, G. Zheng, J. L. Sonnenberg, M. Hada, M. Ehara, K. Toyota, R. Fukuda, J. Hasegawa, M. Ishida, T. Nakajima, Y. Honda, O. Kitao, H. Nakai, T. Vreven, J. A. Montgomery, Jr., J. E. Peralta, F. Ogliaro, M. Bearpark, J. J. Heyd, E. Brothers, K. N. Kudin, V. N. Staroverov, R. Kobayashi, J. Normand, K. Raghavachari, A. Rendell, J. C. Burant, S. S. Iyengar, J. Tomasi, M. Cossi, N. Rega, J. M. Millam, M. Klene, J. E. Knox, J. B. Cross, V. Bakken, C. Adamo, J. Jaramillo, R. Gomperts, R. E. Stratmann, O. Yazyev, A. J. Austin, R. Cammi, C. Pomelli, J. W. Ochterski, R. L. Martin, K. Morokuma, V. G. Zakrzewski, G. A. Voth, P. Salvador, J. J. Dannenberg, S. Dapprich, A. D. Daniels, O. Farkas, J.B. Foresman, J. V. Ortiz, J. Cioslowski, D. J. Fox, Gaussian 09 A.02, Gaussian, Inc., Wallingford, CT, USA, **2009**. 512

[S10] a) J. W. Ochterski, G. A. Petersson, and J. A. Montgomery Jr., *J. Chem. Phys.* **1996**, *104*, 2598–2619; b) J. A. Montgomery Jr., M. J. Frisch, J. W. Ochterski G. A. Petersson, *J. Chem. Phys.* **2000**, *112*, 6532–6542.

[S11] a) L. A. Curtiss, K. Raghavachari, P. C. Redfern, J. A. Pople, *J. Chem. Phys.* **1997**, *106*, 1063–1079; b) E. F. C. Byrd, B. M. Rice, *J. Phys. Chem*. A **2006**, *110*, 1005–1013; c) B. M. Rice, S. V. Pai, J. Hare, *Comb. Flame* **1999**, *118*, 445–458.

[S12] P. J. Lindstrom, W. G. Mallard (Editors), NIST Standard Reference Database Number 69, http://webbook.nist.gov/chemistry/ (accessed June **2020**).

[S13] M. S. Westwell, M. S. Searle, D. J. Wales, D. H. Williams, *J. Am. Chem. Soc*. **1995**, *117*, 5013–5015; b) F. Trouton, *Philos. Mag*. **1884**, *18*, 54–57.

[S14] a) H. D. B. Jenkins, H. K. Roobottom, J. Passmore, L. Glasser, *Inorg. Chem.***1999,** *38,* 3609–3620; b) H. D. B. Jenkins, D. Tudela, L. Glasser, *Inorg. Chem.* **2002**, *41*, 2364–2367.

[S15] P. Banerjee, E. Kemmler, M. Dunkel, R. Preissner, *Nucleic Acids Res.* **2024**, *52*, 513-520.

[S16] ProTox 3.0. Available online: https://tox.charite.de/protox3/ (accessed on 20.06.2025).
